# Transcriptomic analysis links hepatocellular carcinoma (HCC) in HZE ion irradiated mice to a human HCC subtype with favorable outcomes

**DOI:** 10.1038/s41598-021-93467-3

**Published:** 2021-07-07

**Authors:** Liang-Hao Ding, Yongjia Yu, Elijah F. Edmondson, Michael. M. Weil, Laurentiu M. Pop, Maureen McCarthy, Robert L. Ullrich, Michael D. Story

**Affiliations:** 1 Department of Radiation Oncology, The University of Texas Southwestern Medical Center, Dallas, TX 75390 USA; 2 Department of Radiation Oncology, The University of Texas Medical Branch, Galveston, TX 77555 USA; 3 Frederick National Laboratory for Cancer Research, Frederick, MD 21702 USA; 4 Department of Environmental and Radiological Health Sciences, Colorado State University, Fort Collins, CO 80523 USA; 5 NASA Johnson Space Center, Houston, TX 77058 USA; 6 Radiation Effects Research Foundation, Hiroshima, Japan; 7 Harold C. Simmons Comprehensive Cancer Center, The University of Texas Southwestern Medical Center, Dallas, TX 75390 USA

**Keywords:** Cancer, Cancer genomics, Risk factors, Oncogenesis

## Abstract

High-charge, high-energy ion particle (HZE) radiations are extraterrestrial in origin and characterized by high linear energy transfer (high-LET), which causes more severe cell damage than low-LET radiations like γ-rays or photons. High-LET radiation poses potential cancer risks for astronauts on deep space missions, but the studies of its carcinogenic effects have relied heavily on animal models. It remains uncertain whether such data are applicable to human disease. Here, we used genomics approaches to directly compare high-LET radiation-induced, low-LET radiation-induced and spontaneous hepatocellular carcinoma (HCC) in mice with a human HCC cohort from The Cancer Genome Atlas (TCGA). We identified common molecular pathways between mouse and human HCC and discovered a subset of orthologous genes (mR-HCC) that associated high-LET radiation-induced mouse HCC with a subgroup (mrHCC2) of the TCGA cohort. The mrHCC2 TCGA cohort was more enriched with tumor-suppressing immune cells and showed a better prognostic outcome than other patient subgroups.

## Introduction

The uncertainty of cancer risk associated with space radiation is a major concern for human extraterrestrial expeditions^1^. Galactic Cosmic Rays (GCRs) in deep space environments comprise high-charge, high-energy (HZE) ions characterized by high linear energy transfer (high-LET), which contrasts with low-LET terrestrial radiation types, such as photons or γ-rays^2^. Due to the differences in their physical characteristics, high-LET ion radiations induce more complex and persistent DNA damages and are more efficient at cell killing and cell transformation than low-LET radiations. It remains unclear whether these differences at the molecular and cellular levels affect carcinogenic processes and result in distinct molecular and biological features in high-LET radiation-induced tumors.

Significant progress has been made in addressing whether there are qualitative differences between the carcinogenic effects of high- and low-LET radiations. A recent publication using genetically diverse outbred mice demonstrated that the histotype spectrum of HZE ion-induced tumors is similar to the spectra of spontaneous and γ-ray-induced tumors and that the genomic loci that control susceptibility overlap between groups for some tumor types^3^. These results suggest that cancer models and epidemiological data from γ-ray exposures could be used to predict cancer risk from GCRs. The question remains whether the data obtained from mice are applicable to humans, since most research on GCRs’ biological impact has been performed using animal models. A direct comparison of mouse models with human diseases will provide valuable information to improve prediction of carcinogenesis risk and to develop countermeasures against space radiation. Since most genes are orthologous and constitute common signaling pathways between mice and humans, a genomic comparison of cancers between species is a conceivable approach. Indeed, previous data have demonstrated that gene expression signatures associated with clinical and molecular characteristics are conserved in mice and humans^4–6^.

Liver cancer is the second most common cause of death from cancer worldwide. Hepatocellular carcinoma (HCC) is the predominant form of liver cancer^7, 8^. The Cancer Genome Atlas (TCGA) has collected a large number of HCC patients for genomic studies. Substantial molecular classification studies have sought to identify robust subclasses with prognostic implications that could influence the clinical management of HCC. Although some subtyping methods have identified groups with prognostic differences in legacy HCC datasets, none of these classifications has shown significant survival differences in the TCGA HCC cohort^9^.

The radiation risk for causing liver cancer was first reported in atomic bomb survivors^10, 11^. Although it remained controversial that exposure to low-LET radiation was associated with liver carcinogenesis^12^, there was sufficient evidence supporting that high-LET radiation increased liver cancer incidence^13–15^. HCC is one of the major tumor types that high-LET radiation induces in mouse models. Previous analyses using genetically diverse outbred mice have suggested a moderate increase in HCC incidence after high-LET radiation when compared to γ-ray radiation^3^. Other studies using inbred mouse strains susceptible to HCC, i.e*.*, CBA and C3H, have shown a significantly higher incidence of HCC in high-LET-irradiated mice, with a substantial relative biological effectiveness (RBE > 50), than in low-LET-irradiated mice. The dose response curve was consistent with other high-LET-induced solid tumor mouse models^16^. As an extension of the effort to characterize radiation-induced HCC at the molecular level, we have collected tumor and normal liver tissues from mice irradiated with high-LET, γ-ray, or sham radiation, and we have profiled their gene expressions. Comparing molecular features in these HCC samples revealed the underlying mechanisms associated with carcinogenesis induced by high- and low-LET radiation. By using comparative genomics approaches, we have also compared the mouse model of radiation-induced HCC with TCGA human HCC transcriptome profiles to further decipher the molecular mechanisms that are conserved across the two species.

## Results

### Distinct gene expression profiles identified in HCC and non-HCC liver tissues

**Figure 1:**
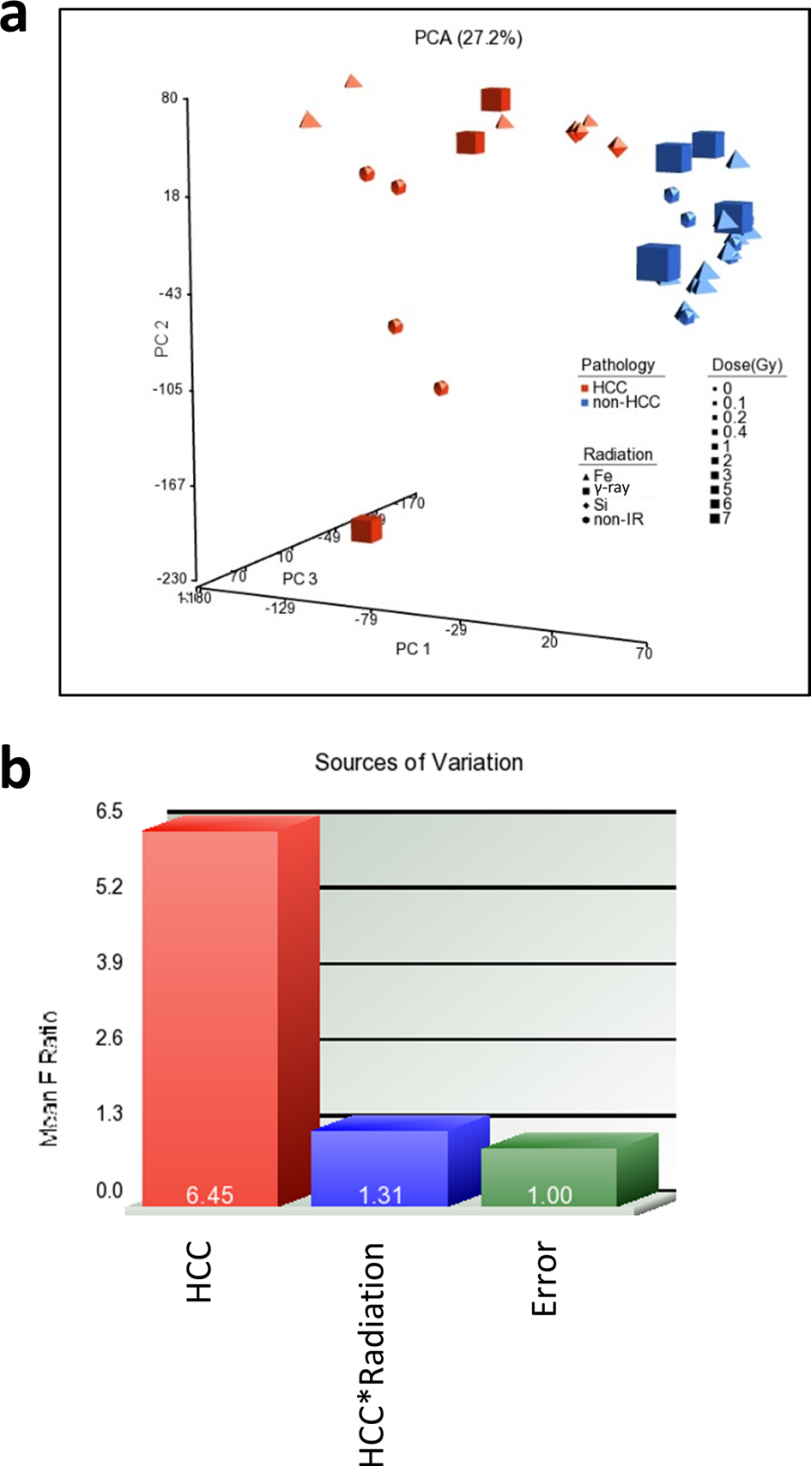
Unsupervised analysis of gene expression profiles in mouse HCC. (**a**) Unsupervised Principle Component Analysis (PCA) indicated distinct gene expression profiles between HCC and non-HCC liver samples. (**b**) Sources of Variation (SOV) analysis showed that HCC contributed to the largest variances. Interactions of HCC and radiation showed a slight increase in F ratios, which indicates modest differences in HCC induced by different radiation types.

We performed gene expression profiling to investigate the molecular features of radiation-induced HCC, especially those induced by high-LET radiations in mouse models. The high-LET irradiations were performed using ^56^Fe and ^28^Si ion particles; the low-LET irradiations were performed using γ-rays. All the radiation types consisted of different doses (Supplementary Table S1). We also included age-matched non-HCC liver samples from ^56^Fe and γ-rays-irradiated mice, and normal livers from sham-irradiated mice as control groups. Unsupervised Principal Component Analysis (PCA) suggested distinct gene expression profiles between HCC and non-HCC liver tissues regardless of whether the mice were irradiated or not (Fig. 1a). This is further evidenced by the source of variation analysis (SOV), which showed that tumors contribute to the largest mean F ratios, more than sixfold above background noise. Although samples did not separate based on radiation type in the PCA analysis, interactions between tumor and radiation in SOV caused a slight increase in F ratios, which indicates that there may be modest differences in HCC induced by different radiation types (Fig. 1b).

### Differentially expressed genes drive changes in signaling pathways in different types of mouse HCC

**Figure 2:**
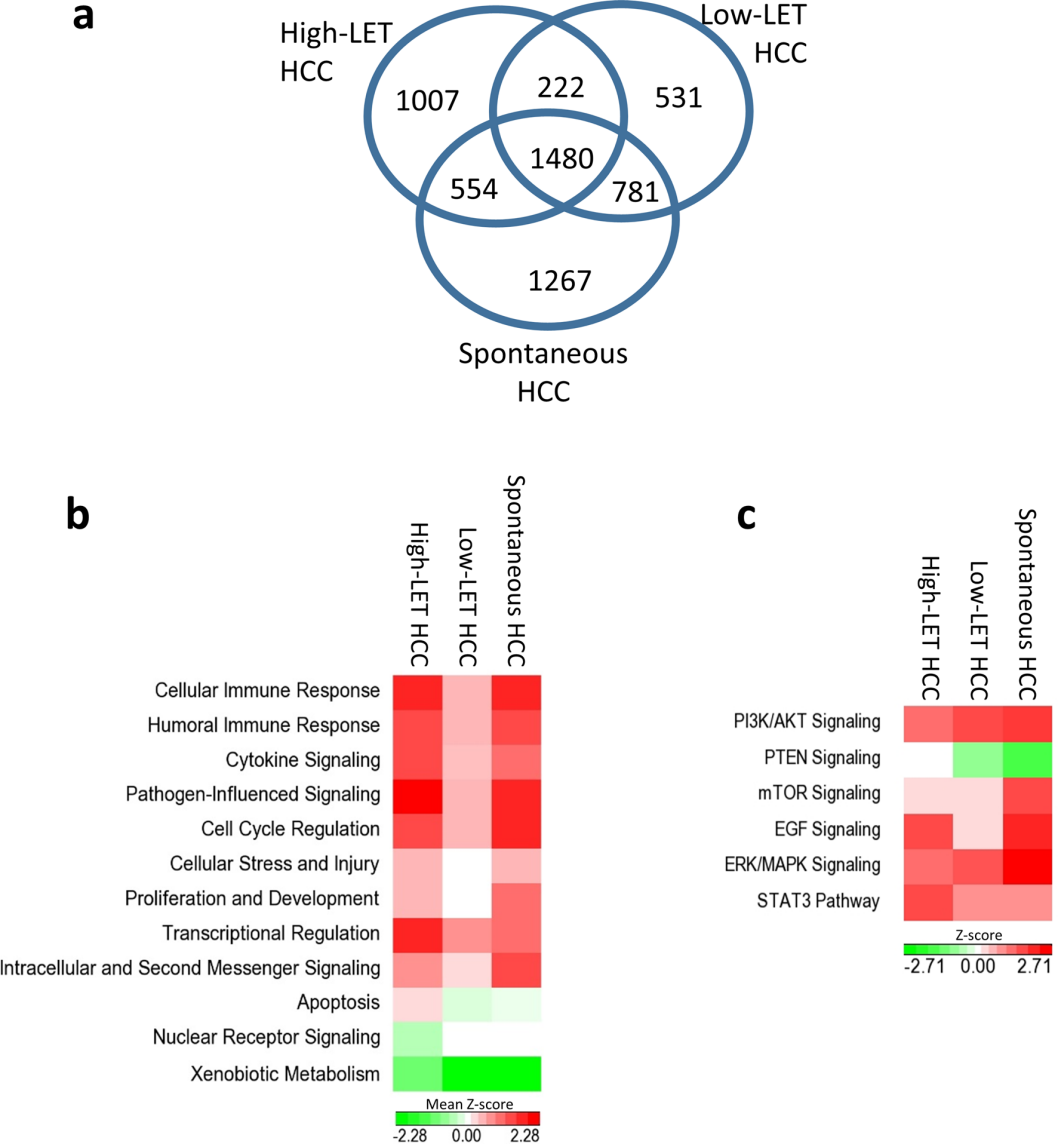
Differential gene expression and signaling pathways analysis. (**a**) Venn diagram shows the number of differentially expressed genes in different types of mouse HCC in comparison with normal mouse liver. (**b**) Canonical signaling pathways that changed in each type of mouse HCC were summarized into functional groups. The heatmap shows averaged z-scores of all pathways within each functional category. A positive z-score indicates activation; a negative z-score indicates suppression. (**c**) Signaling pathways known to be associated with human HCC also changed in mouse HCC models. Z-scores representing activation (positive) or suppression (negative) of the pathways were shown as a heatmap.

We performed differential gene expression analysis in high-LET radiation-induced HCC, low-LET radiation-induced HCC and spontaneous HCC by using a two-way ANOVA model to compare against baseline expression in non-irradiated normal livers. The statistical power of this model was greater than 80% at 10% of false discovery rate (FDR). We identified significant numbers of genes that were differentially expressed in each type of HCC (Fig. 2a, Supplementary Table S2). Ingenuity Pathway Analysis showed that these genes were associated with signaling pathways, which we grouped into 12 categories based on their biological functions. Averaged z-scores represented levels of activation or suppression for signaling pathways in each functional category (Fig. 2b). Most of the functional categories were either upregulated in all three types of HCC or downregulated in all three types of HCC, though the z-scores representing the magnitudes of up/downregulations differed. Signaling pathways under each category and their z-scores are summarized in the supplementary data. Pathways under three categories, i.e., Cellular Immune Response, Humoral Immune Response and Cytokine Signaling, comprised a large proportion (38%) of all pathways changed (Supplementary Table S3A). Pathways involved in reactive oxygen species production and in xenobiotic metabolisms were among the most activated and most inhibited signaling pathways, respectively, according to z-scores (Supplementary Table S3B). Canonical pathways known to be altered in human HCC were also changed in mouse HCC. These included the Pi3k, Pten, mTor, Egf, Erk/Mapk and Stat3 signaling pathways. Z-scores of the activated Pi3k signaling and the suppressed Pten signaling pathways were inversely correlated (Fig. 2c).

### Supervised clustering identified genes (mR-HCC) associated with radiation effects in mouse HCC

**Figure 3:**
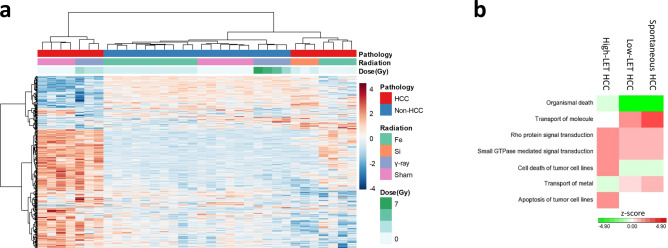
A gene set (mR-HCC) that discriminated mouse HCC induced by different radiation types. (**a**) Supervised clustering analysis using genes identified from ANOVA not only clustered HCC and non-HCC samples, they also separated un-irradiated and different radiation types in each group. (**b**) Gene functional analysis showed significantly enriched functional groups associated with the gene set. The heatmap was plotted with activation z-scores.

We obtained a gene set (mR-HCC) containing 357 Illumina probes (280 known mouse genes) by applying FDR < 0.2 to the interactive term of radiation type and HCC in the two-way ANOVA model. Clustering analysis of mR-HCC genes separated the sample cohort into non-HCC liver, high-LET radiation-induced HCC and low-LET/spontaneous HCC. Within the non-HCC group, mR-HCC genes separated samples by radiation type. Low-LET and spontaneous HCC showed similar expression patterns that differed from high-LET radiation-induced HCC and non-HCC samples (Fig. 3a).

Functional analysis suggested that mR-HCC was enriched with genes associated with organismal and cell death/apoptosis, as well as with molecular transport, Rho signaling and small GTPase-mediated signaling transduction (Fig. 3b). High-LET-induced HCC showed increased signaling (higher z-scores) in tumor cell death/apoptosis, whereas low-LET/spontaneous HCC showed higher z-scores in molecular transport, which suggests diverse activities of gene functions in different types of HCC. Detailed functional analysis results are listed in supplementary data (Supplementary Table S4A).

### The mR-HCC genes identified novel human HCC subtypes associated with radiation-induced HCC in mice

**Figure 4:**
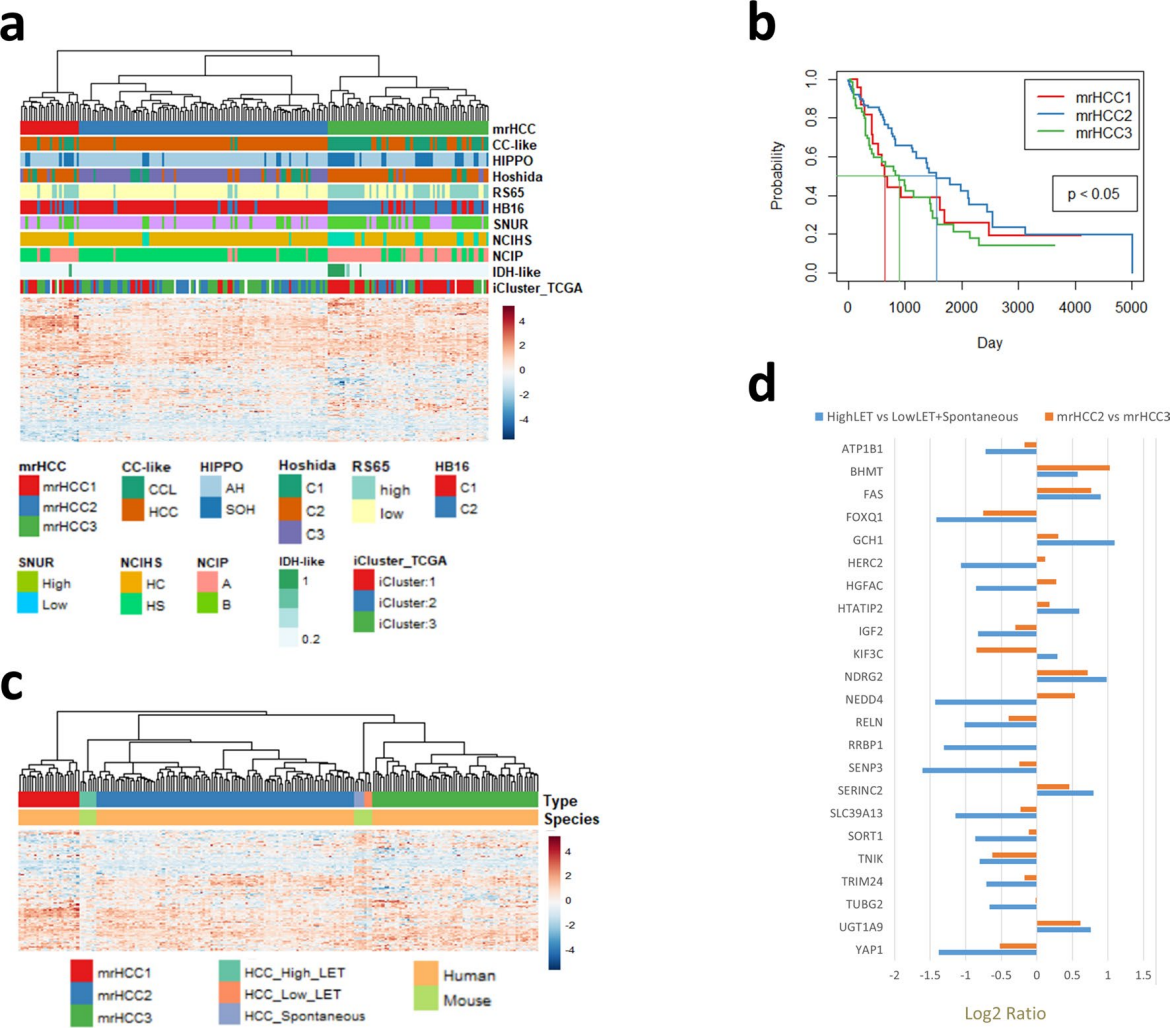
The mR-HCC gene set derived from the mouse HCC model identified subgroups of the human TCGA HCC cohort. (**a**) Clustering analysis of TCGA HCC samples using log2 ratios of mR-HCC genes. The clustering defined 3 clusters: mrHCC1, mrHCC2, and mrHCC3. Rows of annotations under the dendrogram are overlaid with molecular subtypes previously published by TCGA. (**b**) Kaplan–Meier analysis in human HCC based on mrHCC clusters. The survival probability differed significantly (p < 0.05) across the three clusters, with patients in the mrHCC2 subgroup showing better survival. Straight lines indicate median survival time in each mrHCC subgroup. (**c**) Clustering analysis of integrated mouse and human expression data using mR-HCC genes. High-LET radiation-induced mouse HCC samples were clustered in mrHCC2; low-LET radiation-induced and spontaneous mouse HCC were clustered in mrHCC3. Heatmap was plotted using log2 radios of gene expressions between tumor and normal liver. (**d**) Comparison of averaged expression ratios using a subset of mR-HCC genes identified as related to HCC via data mining of the published literature. The mouse expression ratios were high-LET vs. low-LET/spontaneous HCC samples; the human expression ratios were mrHCC2 vs. human mrHCC3 samples.

By using mouse and human gene orthologue mapping, we matched 260 genes within mR-HCC genes that overlap with the TCGA human HCC RNA-seq dataset (Supplementary Table S4B). The mR-HCC genes identified three subgroups in the human TCGA cohort by clustering analysis, which we named mrHCC1, mrHCC2 and mrHCC3 (Fig. 4a).

We overlaid mrHCC clusters with other molecular subtypes and clinical features published by TCGA^9^ and performed Chi-square/Krustal–Wallis tests to identify correlations. Detailed results are summarized in Fig. 4a and in the supplemental data (Supplementary Table S5). Among significantly correlated molecular subtypes, mrHCC1 and mrHCC3 exhibited similarity to the cholangiocarcinoma-like (CCL-like) signature subtype, silencing of the Hippo pathway (Hippo), non-differentiated (Hoshida C2), high risk score (RS65) and high-proliferating (HB16, SNUR and NCIP) phenotypes; whereas mrHCC2 was enriched with HCC-like signature subtype, activation of Hippo pathway, well-differentiated (Hoshida C3), low risk score and low-proliferating phenotypes. Most of the hepatic stem cell subtypes (HS) were clustered in mrHCC3 and mrHCC1, but significantly less in mrHCC2, based on hepatic stem cell signature (NCIHS). Isocitrate dehydrogenase isozyme (IDH)-like subtype was identified by TCGA based on IDH1/2 mutation signature and was correlated with poor prognosis. All IDH-like samples were clustered in mrHCC3 and mrHCC1; none were clustered in mrHCC2. Integrated clusters (iCluster1, iCluster2 and iCluster3) were identified by TCGA using a joint multivariate regression approach to simultaneously cluster data from five platforms (DNA copy number, DNA methylation, mRNA expression, miRNA expression and RPPA). Compared with iCluster2 and iCluster3, iCluster1 showed higher tumor grade, presence of macrovascular invasion, poorly differentiated samples, and overexpression of proliferation marker genes. iCluster1 made up 61.9% of the mrHCC3 and 54.5% of the mrHCC1 samples, but only 13.4% of the mrHCC2 samples. Over 86% of the mrHCC2 samples were from iClusters 2 and 3.

Despite the IDH-like subtype and iCluster1 overlapping with features correlated with poor prognosis, there was no difference in overall survival based on the IDH or iCluster groups in the TCGA cohort^9^. Kaplan–Meier analysis across the three mrHCC clusters indicated that the mrHCC2 cluster showed a better survival probability than mrHCC1 or mrHCC3 (p < 0.05) (Fig. 4b). The median survival time was 643 days for mrHCC1, 1560 days for mrHCC2 and 899 days for mrHCC3. These results suggest that mrHCC clusters exhibited better correlation with clinical outcome of HCC patients than the TCGA iClusters.

To compare mouse HCC with human HCC subtypes, we integrated expression data of mR-HCC genes using log2 ratios of HCC vs. normal liver gene expression in the TCGA RNA-seq data and in mouse HCC expression analysis. High-LET-induced mouse HCC samples were clustered with mrHCC2 in human HCC. Spontaneous and low-LET-induced mouse HCC samples were clustered with mrHCC3 (Fig. 4c). We identified 23 genes from the mR-HCC signature that were known to be associated with HCC via data-mining from published literature^17–37^. We compared the expression ratios between high-LET and low-LET/spontaneous mouse HCC with human gene expression ratios between mrHCC2 and mrHCC3 samples. Most of the genes (18 of 23) showed consistent up- or downregulation between mouse and human samples (Fig. 4d). This supported our findings from the clustering analysis that high-LET-induced mouse HCC was similar to human mrHCC2 samples and that low-LET-induced HCC was similar to human mrHCC3 samples.

### Profiles of tumor-infiltrating immune cells in subtypes of mouse and human HCC

**Figure 5:**
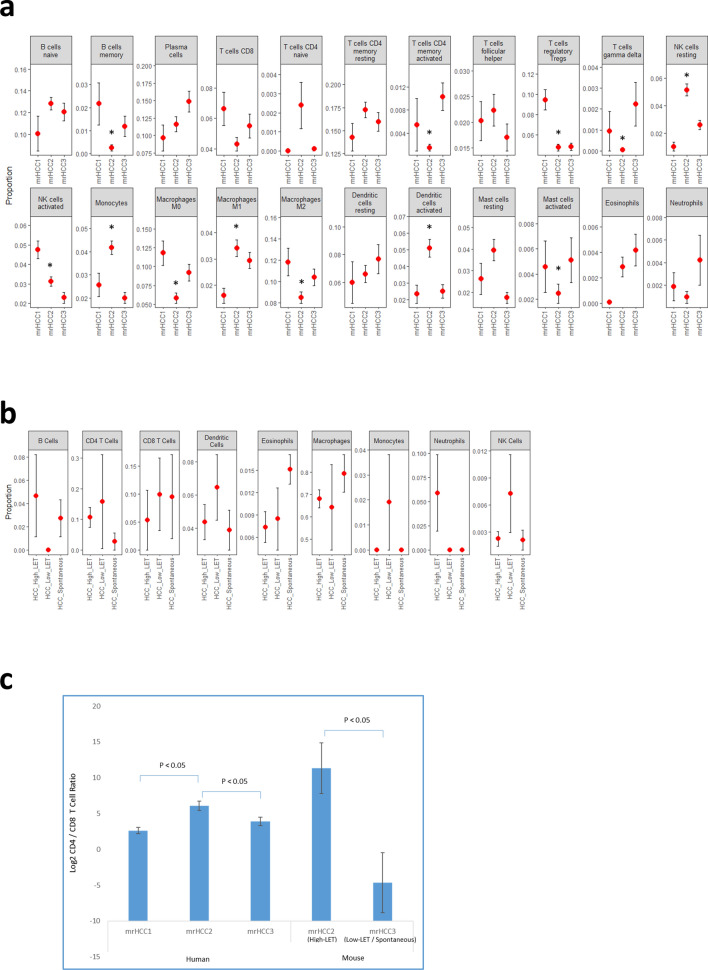
Profiles of tumor-infiltrating immune cells in mouse and human HCC. (**a**) Average values of relative proportions of immune cells in each mrHCC cluster of human TCGA HCC cohorts. Bar: standard error. *Immune cell proportions of mrHCC2 were significantly different from either mrHCC1 or mrHCC3 (p < 0.05). (**b**) Average values of relative proportions of immune cells in high-LET, low-LET and spontaneous mouse HCC. Bar: standard error. (**c**) Ratios of CD4+/CD8+ tumor-infiltrating T cells in different subgroups of mouse and human HCC. Bar: standard error.

We characterized the immune microenvironments in mouse and human HCC by profiling immune infiltrates using ImmuCC or CIBERSORTx to infer the relative abundances of different immune cell types. ANOVA results suggested that several immune cell types differed significantly across mrHCC subtypes in human HCC (Fig. 5a). Among these, macrophage subtypes M1 and M2 and T regulatory (Treg) lymphocytes are predictive immunocytes of HCC prognosis. Our analysis showed that high levels of M1 and low levels of M2 and Treg in mrHCC2 samples indicated better survival and were consistent with the Kaplan–Meier analysis. Although profiles of mouse HCC immune infiltrates suggested changes associated with radiation types, none of the differences were statistically significant (Fig. 5b). Previous studies showed that the CD4+/CD8+ T cell ratio was positively correlated with the survival of patients with cancer ^38, 39^. We further investigated the CD4+/CD8+ ratio in mouse and human samples and found that mrHCC2 samples, whether from mouse models or the human HCC cohort, have significantly higher ratios than mrHCC1 and mrHCC3 (p < 0.05) (Fig. 5c).

## Discussion

Data from recent publications showed no differences in the spectrum of tumors induced by high-LET or low-LET radiations in a genetically diverse outbred mouse cohort, which suggests shared mechanisms of tumorigenesis regardless of the type of radiation exposure^3^. This agrees with our pathway analysis findings in radiation-induced mouse HCC models. Canonical signaling pathways associated with HCC showed the same activation/suppression status in tumors induced by both high-LET and low-LET radiation. Inflammatory response, including cellular immune signaling and cytokine signaling accounted for the largest portion among all pathways. Reactive oxygen species production and xenobiotic metabolism pathways were the most activated and the most suppressed pathways, respectively, probably due to the impaired liver functions. The pathway analysis results also made it possible to compare the molecular mechanisms of mouse tumorigenesis with those of human HCC. The PTEN signaling pathway is a tumor suppression pathway whose downregulation leads to hyperactivation of the PI3K/AKT/mTOR signaling pathway in human HCC^40, 41^. Pten, Pi3k/Akt and mTor signaling showed strong associations with all types of HCC in the mouse model. Activation z-scores of the Pi3k/Akt pathway were inversely correlated in different types of mouse HCC, which suggests analogous molecular mechanisms between mouse and human tumors. ERK/MAPK and EGF signaling are other pathways associated with human HCC^42^. Both of these pathways were activated in all types of mouse HCC, with upregulation of downstream genes such as c-Fos and Pkcα.

Although the common pathways involved in HCC induced by low-LET and high-LET radiation indicated similar molecular mechanisms, the variations in z-scores for each pathway suggested differences in the levels of activation or suppression associated with each type of HCC. These quantitative differences may result in different biological characteristics in low-LET and high-LET-induced cancer. Using supervised ANOVA, we identified a gene set (mR-HCC) that discriminated the HCC from the non-HCC mouse liver based on the types of radiation exposure. Functional analysis of the mR-HCC gene set suggested higher apoptosis and cell death activities in high-LET-induced HCC. This is mechanistically consistent with our findings that HCC induced by high-LET radiation had a tumor-suppressing immune cell profile and clustered with the human HCC subgroup that had better survival.

Previous study has shown that the incidence of Fe and Si ion radiation-induced HCC followed the same dose–response curve in mouse model. The HCC incidence peaked at 0.2 Gy for both Fe and Si, but only started to increase after 2 Gy of γ-ray irradiation^16^. The different dose ranges between the high- and low-LET radiations in our experiment were selected to maximize the tumor incidence. Nonetheless, it is notable that mR-HCC genes separated HCC samples induced by Fe and Si ions within the high-LET radiation group. Unfortunately, due to the limited sample size, the statistical power decreased significantly when we un-pooled the high-LET HCC samples into two groups (Supplementary Fig. S1). Therefore, we did not perform differential expression analysis to interrogate the differences between the two ions. Activation z-scores of signaling pathways calculated separately based on Fe and Si radiations showed similarities of the two ions and consistent with the results of the high-LET HCC samples as a group (Supplementary Fig. S2A,B). Gene ontology of the mR-HCC genes showed consistent trend of higher apoptosis and cell death activities in both Fe and Si groups (Supplementary Fig. S2C).

The mR-HCC gene set allowed us to directly compare expression profiles between the TCGA human HCC cohort and the mouse HCC model. We identified three novel subgroups, mrHCC1, mrHCC2 and mrHCC3, by clustering analysis using human HCC expression data in TCGA. The human mrHCC subgroups demonstrated significant differences in overall survival, with mrHCC2 showing better prognosis than mrHCC1 and mrHCC3. The median survival time of mrHCC2 was more than 600 days longer than the other subgroups. The high-LET radiation-induced mouse HCC showed a similar expression profile to the mrHCC2 cluster; the low-LET and spontaneous mouse HCC showed similar profiles to the mrHCC3 cluster. The consistency of the expression ratios of known HCC-associated genes between mrHCC2 vs. mrHCC3 in humans and high-LET vs. low-LET/spontaneous HCC in mice further supported the clustering results.

We characterized the mrHCC clusters by finding overlapping molecular and clinical features in the human HCC cohort published by TCGA. The most significant overlaps were in features already identified by other genomics clustering analyses or by historic gene signatures associated with HCC, which suggests that the molecular mechanisms of human HCC resemble those in the mouse model. Among the overlapping features, two gene signatures revealed the biggest differences between mrHCC/high-LET and mrHCC3/low-LET/spontaneous HCC samples. The CCL and NCIHS signatures showed that the mrHCC3/low-LET/spontaneous group was more cholangiocarcinoma-like (67%) and positive for the hepatic stem cell signature (33%), whereas mrHCC2/high-LET samples were more HCC-like (97%) and positive for the hepatocyte signature (96%). Many other features, though lacking statistical significance, showed associations with particular mrHCC clusters (Supplementary Table S5). It is noteworthy that none of the published features from TCGA identified subgroups with different prognoses in the TCGA HCC cohort, whereas the mrHCC clusters showed significant differences in overall survival.

The dualistic role of the immune system in HCC carcinogenesis resides on the balance between cancer-promoting inflammation and immune surveillance that hinders tumor development^43^. The distribution and the networking of tumor infiltrating immune cell populations are the key players in the tumor-immune ecosystem. Computational tools such as CIBERSORTx de-convolute cell types by using immune cell signature gene expression to estimate the global immune cell composition from whole tumor gene expression data. Immune cell profiling of mrHCC clusters in human samples suggested that more than half of the cell types (12 of 23) differed significantly across the clusters (FDR < 0.05). When comparing the antigen presenting cell (APCs) phenotype of the mHCC2 TCGA cohort immune phenotype to mrHCC1 and mrHCC3, we identified several significant differences. mrHCC2 showed a higher intratumoral infiltration of activated dendritic cells (DCs), naïve B cells, monocytes and anti-tumor M1 macrophages, while pro-tumor M2 macrophages where at the lowest relative proportion. The mrHCC2 subgroup exhibited a higher M1/M2 ratio, which is known to be associated with better survival^44^. The relative proportion of HCC-promoting immune cells, such as neutrophils^45^, activated mast cells^46^, B cells and plasma cells^47^, was abated only in the mrHCC2 subgroup. The analysis of CD4 + T cell subsets reveals for the mrHCC2 subgroup a minimal immune suppressive Treg cell profile, with a maximal naïve, memory resting and follicular helper cells signature, suggesting a robust control of anti-tumor immune responses^48^. Moreover, our data support recent data which show that the intratumoral selective accumulation of resting memory CD4+ T cells at the expense of memory activated CD4+ T cells denotes a pivotal mechanism to constrain tumor growth^49^. Finally, despite a low relative proportion of CD8+ T cells, the CD4+/CD8+ T cell ratio was found at the highest amplitude in the mrHCC2 subgroup, confirming previous studies where a similar trend was observed only in patients with HCC who had better prognosis^50, 51^. It has also been shown that the CD4+/CD8+ T cell ratio is a more consistent prognostic marker than CD8+ cells alone^38, 39^. Mouse data of tumor infiltrating immune cells indicated a significantly higher CD4+/CD8+ cell ratio in high-LET HCC than low-LET/spontaneous HCC, though individual mouse immune cell types showed bigger variations but no statistically significant differences, probably due to small sample sizes.

Even though high-LET-induced HCC samples were clustered with the human subgroup that demonstrated favorable survival outcomes, we do not suggest that high-LET radiation is safer than terrestrial-originated radiations. Mouse epidemiological data indicate that high-LET radiation induces a higher incidence of liver tumors, and the median survival in the TCGA HCC mrHCC2 cohort is only 4.2 years^9^. Our comparative transcriptome analysis classified high-LET radiation-induced HCC into human HCC subgroups with unique molecular features and will help further mechanistic studies and countermeasure development for space radiation. The fact that high-LET radiation induced more tumor-suppressing immune cells was consistent with previous studies showing that immunotherapy plus heavy ion particle radiotherapy, not photon radiation, increased antitumor immunity in mouse cancer models^52–54^, which suggests a potential therapeutic advantage for cancer radiotherapy that uses heavy ion particle beams.

## Methods

### Mice and radiations

The details of the mouse carcinogenesis study covering this project have been described previously^16^. All animal procedures were conducted under protocols approved by the Institutional Animal Care and Use Committee at the University of Texas Medical Branch (UTMB) and Brookhaven National Laboratory (BNL). All experiments were performed in accordance with relevant guidelines and regulations. The study was reported in accordance with ARRIVE guidelines. Briefly, 8- to 10-week-old male C3H/HeNCrl mice were purchased from Charles River Laboratories and shipped to the Brookhaven National Laboratory (BNL). After a 2–3 day acclimation, these mice were subjected to single-dose whole-body irradiation with either 600 MeV/n ^56^Fe ions (LET = 181 keV/µm) or 300 MeV/n ^28^Si ions (LET = 64 keV/µm), both at a dose range of 0.1–1.0 Gy, and ^137^Cs gamma rays at a dose range of 1–3 Gy. Control mice were sham-irradiated at BNL under the same conditions as the irradiated groups. The mice were not anesthetized during irradiation nor in any part of the study. After a 3–5 day resting period after irradiation, mice were shipped to the Animal Resource Center at UTMB, where they were housed 5 per cage in ventilated cage racks using Harlan Sani-chip bedding and maintained on a Harlan 7912 diet and autoclaved reverse osmosis purified water ad libitum throughout the study. Animals were monitored daily and palpated weekly. They were euthanized when moribund and/or displaying signs and symptoms of liver tumors or other neoplasms or when they reached 800 days of age. Dead, moribund, or symptomatic animals were necropsied, and tissues (including lung, liver, spleen) were harvested for histological evaluation and storage in a – 80 °C freezer after being snap frozen in liquid nitrogen. All necropsies and histological examinations were performed blinded. Liver tumors were confirmed using standard H&E histopathology and classified as hepatocellular carcinoma (HCC) or liver adenomas.

### Total RNA extraction from tissues

Tumors or normal liver tissues were homogenized in Qiazol reagent (Qiagen, Inc.), and total RNA was extracted by using miRNeasy Mini Kit (Qiagen, Inc.) according to the manufacturer’s protocol. RNA quality was checked by measuring absorbance ratios of 260/280 nm and 260/230 nm via a Nanodrop spectrophotometer (ThermoFisher Scientific).

### Labeling and hybridization of microarray for gene expression analysis

Gene expression profiling was performed by using Mouse Whole Genome Gene Expression BeadChip (Illumina). Samples included 7 high-LET radiation-induced HCC, 3 low-LET radiation-induced HCC, 4 spontaneous HCC, 10 non-HCC livers from high-LET–irradiated mice, 4 non-HCC livers from low-LET–irradiated mice and 6 normal livers from sham-irradiated mice. Each RNA sample, with 500 ng of total RNA, was amplified via the Ambion TotalPrep RNA amplification kit (ThermoFisher Scientific). This kit uses a T7 oligo(dT) primer to generate single-stranded cDNA, then a second strand synthesis to generate double-stranded cDNA, which is then column purified. We used in vitro transcription to synthesize biotin-labeled cRNA via T7 RNA polymerase. We then checked the column-purified cRNA for size and yield by using the Bio-Rad Experion system, and we hybridized 1.5 µg of cRNA to each array by using standard Illumina protocols with streptavidin-Cy3 (Amersham) for detection. Arrays were scanned on an Illumina Beadstation.

### Statistical analysis of cDNA microarray and RNA-seq data

Gene expression signals from Illumina mouse BeadChip were summarized via GenomicStudio 3.0 (Illumina). We background-subtracted and quantile-normalized these summarized data by using the MBCB algorithm^55, 56^. Differential gene expression analysis was performed by implementing a two-way ANOVA model in the Partek Genomics Suite 7.0. The ANOVA model included two main effects, pathological diagnosis and LET of radiation, and an interaction term of the two main effects. Genes were significantly changed when FDR < 0.1 and the fold changes were greater than 1.3. Pathway analysis was performed via Ingenuity Pathway Analysis (IPA) (Qiagen), December 2019 release. Pathways were considered significantly changed when statistical cutoffs of FDR and z-score were met in at least one tumor type. The cutoff of FDR values generated by IPA’s Fisher Exact Test was less than 0.01, and the cutoff of z-scores was greater than 1 for pathway analysis. We downloaded the RSEM expected counts of TCGA and GTEx RNA-seq data from the UC Santa Cruz Treehouse data repository. We used RNA-seq data from a total of 192 HCC and 110 normal liver samples in this study. The count files were processed and normalized by tximport^57^ and DESeq2^58^. We transformed the normalized counts by the vst function in the DESeq2 package. Log2 ratios of each gene were scaled to a standard deviation before comparison analysis. Kaplan–Meier analysis was performed by using the R package “survival.” Mouse and human gene names were matched based on Ensembl orthologue mapping. We profiled infiltrating immune cells in mouse HCC by using the R script provided by the authors of ImmuCC^59^. We profiled the TCGA HCC immune cells by using the web interface of CIBERSORTx^60^. We calculated statistical significances of immune cell abundances across the three mrHCC clusters by ANOVA analysis.

## Supplementary Information

Supplementary Figures.

Supplementary Table S1.

Supplementary Table S2.

Supplementary Table S3A.

Supplementary Table S3B.

Supplementary Table S4A.

Supplementary Table S4B.

Supplementary Table S5.

## Data Availability

Mouse gene expression data are deposited in GEO (GSE166653).
