# Supplementary material for: Transcriptomic analysis links hepatocellular carcinoma (HCC) in HZE ion irradiated mice to a human HCC subtype with favorable outcomes
Source: Sci Rep. 2021 Jul 7;11:14052. doi: 10.1038/s41598-021-93467-3 (PMC8263559; doi:10.1038/s41598-021-93467-3)
Supplement: Supplementary file 1 — Supplementary Figures. [file 41598_2021_93467_MOESM1_ESM.pdf]

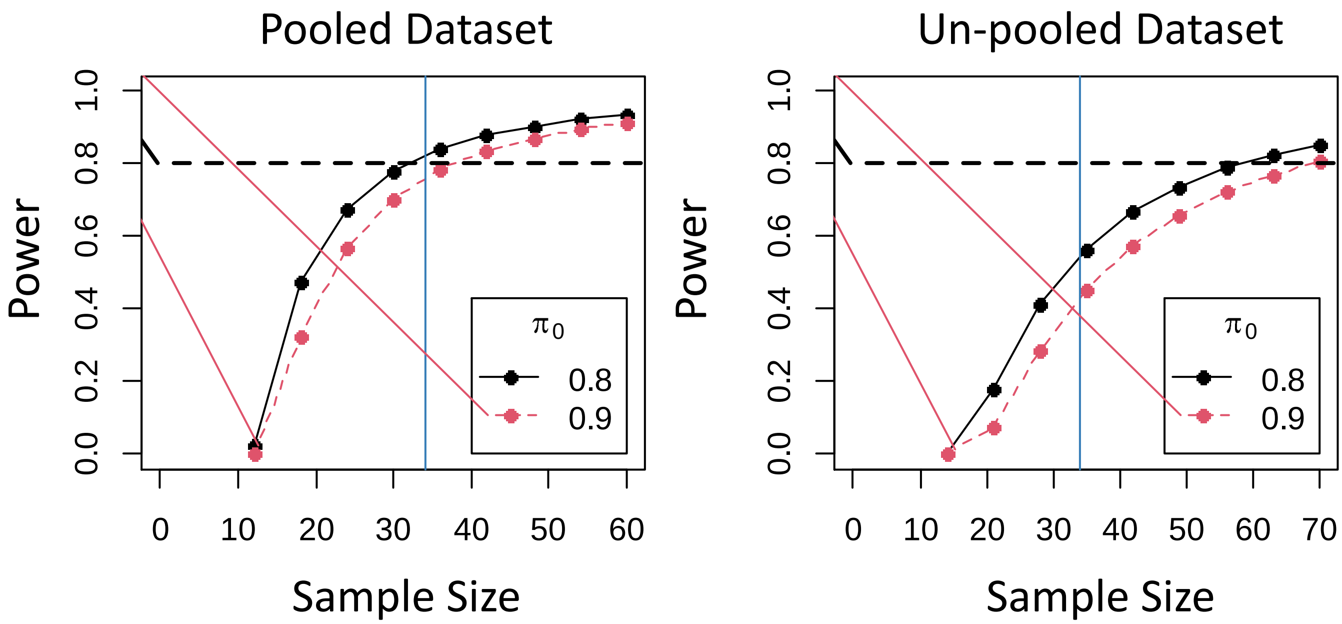

Fig. S1. Power analysis for discovering differentially expressed genes between various types of HCC and normal liver. Samples from Fe and Si radiation were pooled or un-pooled in high-LET radiation group. The analysis was performed with fdr < 0.1 and using proposed algorithm by Liu and Hwang 2007. Vertical line indicated 34 which is the samples size of our dataset. π_0 values are the estimated proportions of genes that are not changed.

Reference:

Liu, P. and Hwang, J.T. (2007) Quick calculation for sample size while controlling false discovery rate with application to microarray analysis. *Bioinformatics*, **23**, 739-746.

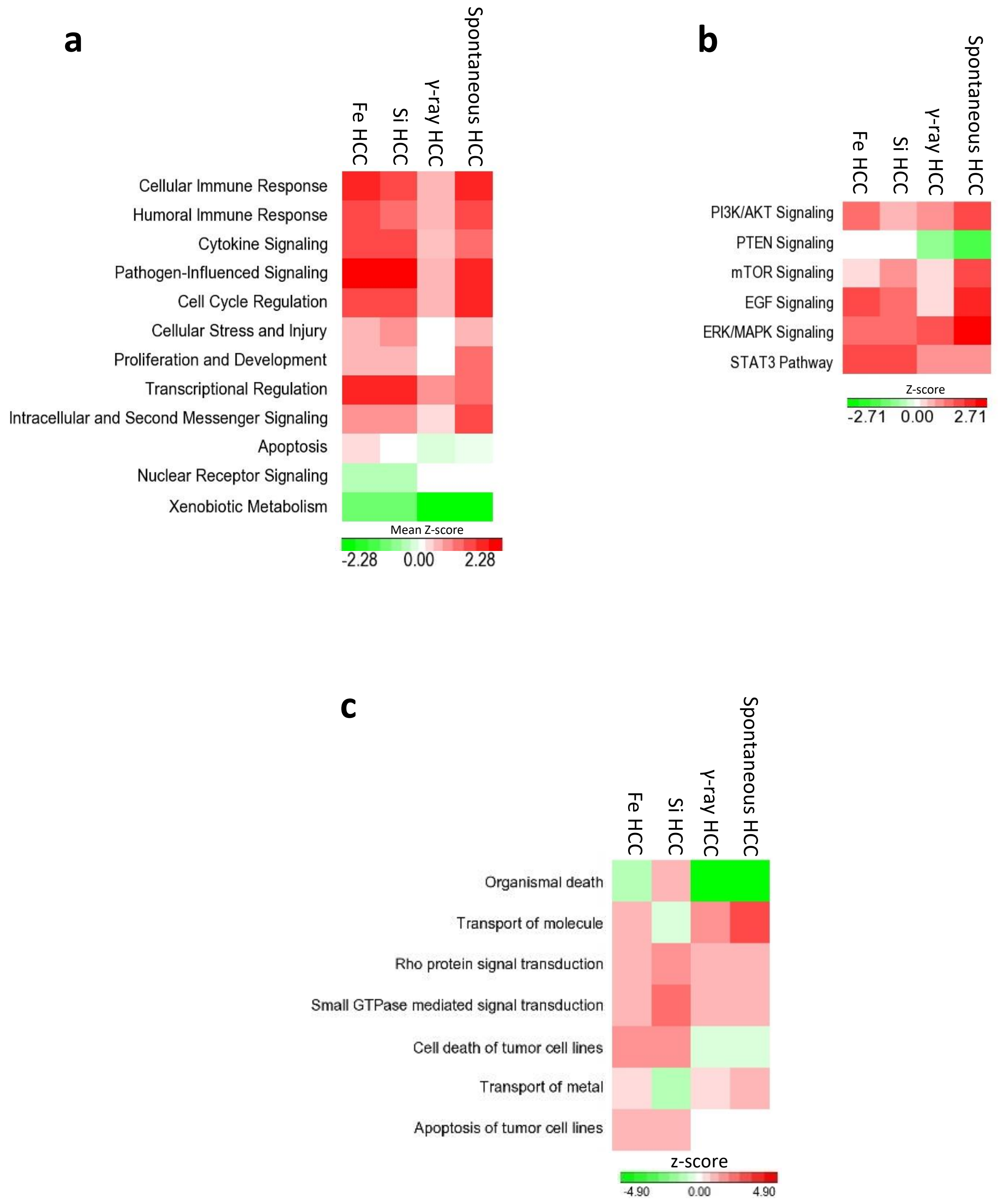

Fig. S2. Z-scores of signaling pathways were plotted as heatmaps from different types of HCC. High-LET HCC samples were divided into Fe and Si induced HCC groups. A. averaged z-scores of all pathways within each functional category. A) positive z-score indicates activation; a negative z-score indicates suppression. B) Signaling pathways known to be associated with human HCC also changed in mouse HCC models. Z-scores representing activation (positive) or suppression (negative) of the pathways were shown as a heatmap. C) Gene functional analysis of mR-HCC genes showed significantly enriched functional groups associated with the gene set.
