# Supplementary material for: Transcriptomic analysis links hepatocellular carcinoma (HCC) in HZE ion irradiated mice to a human HCC subtype with favorable outcomes
Source: Sci Rep. 2021 Jul 7;11:14052. doi: 10.1038/s41598-021-93467-3 (PMC8263559; doi:10.1038/s41598-021-93467-3)
Supplement: Supplementary file 8 — Supplementary Table S5. [file 41598_2021_93467_MOESM8_ESM.pdf]

Table S5. mrHCC Clusters vs TCGA Features

Contents

Molecular Subtyping . . . 1
Clinical Data . . . 3
Hepatitis Calls . . . 10
Leukocyte Estimate . . . 10
Purity/Ploidy . . . 10
Historic Gene Signature . . . 11
TP53 / CTNNB1 / TERT . . . 12
Pathology Review . . . 13
mRNA Fusions . . . 16

Numbers in tables are percent of feature in each mrHCC cluster

Molecular Subtyping

```
RPPA clusters (Rehan Akbani)
Chi-square test p-value: 2.917717e-06
Bonferroni adjusted p-value: 0.0002363351
```

	mrHCC1	mrHCC2	mrHCC3
1	59.09	29.49	72.41
2	40.91	70.51	27.59

```
miRNA clusters (5 group NMF, Robertson group)
Chi-square test p-value: 2.606704e-07
Bonferroni adjusted p-value: 2.11143e-05
```

	mrHCC1	mrHCC2	mrHCC3
#N/A	0.00	2.94	1.52
1	8.33	24.51	13.64
2	25.00	35.29	4.55
3	50.00	16.67	36.36
4	0.00	2.94	21.21
5	16.67	17.65	22.73

```
Hypermethylation Cluster (Laird group)
Chi-square test p-value: 1.224833e-09
Bonferroni adjusted p-value: 9.921146e-08
```

	mrHCC1	mrHCC2	mrHCC3
1	20.83	4.90	27.27
2	25.00	47.06	34.85
3	33.33	3.92	27.27

4	20.83	44.12	10.61

Hypomethylation Cluster (Laird group)
Chi-square test p-value: 0.0001523342
Bonferroni adjusted p-value: 0.01233907

	mrHCC1	mrHCC2	mrHCC3
1	41.67	20.59	46.97
2	41.67	33.33	36.36
3	16.67	46.08	16.67

mRNA clusters (5 group NMF, Hoadley group)
Chi-square test p-value: 2.772859e-22
Bonferroni adjusted p-value: 2.246016e-20

	mrHCC1	mrHCC2	mrHCC3
1	34.78	2.97	75.76
2	13.04	0.99	1.52
3	21.74	21.78	9.09
4	8.70	49.50	1.52
5	21.74	24.75	12.12

DNA copy clusters, version 2 (Cherniak group)
Chi-square test p-value: 0.0003038352
Bonferroni adjusted p-value: 0.02461065

	mrHCC1	mrHCC2	mrHCC3
1	36.36	30.69	18.75
2	18.18	39.60	48.44
3	45.45	13.86	29.69
4	0.00	15.84	3.12

CDKN2A silencing (Toshinori Hinoue)
Chi-square test p-value: 0.1332147
Bonferroni adjusted p-value: 1

	mrHCC1	mrHCC2	mrHCC3
0	34.78	43.56	56.06
1	65.22	56.44	43.94

Paradigm clusters (k=4, Kiley Graim)
Chi-square test p-value: 1.643718e-16
Bonferroni adjusted p-value: 1.331412e-14

	mrHCC1	mrHCC2	mrHCC3
Paradigm:C1	9.09	34.00	12.50
Paradigm:C2	27.27	23.00	20.31
Paradigm:C3	13.64	6.00	65.62
Paradigm:C4	50.00	37.00	1.56

iCluster clusters (k=3, Ronglai Shen)
Chi-square test p-value: 6.378108e-09
Bonferroni adjusted p-value: 5.166268e-07

	mrHCC1	mrHCC2	mrHCC3
iCluster:1	54.55	13.40	61.90
iCluster:2	18.18	42.27	15.87
iCluster:3	27.27	44.33	22.22

Clinical Data

Status
Chi-square test p-value: 0.1138658
Bonferroni adjusted p-value: 1

	mrHCC1	mrHCC2	mrHCC3
Alive	37.50	55.88	42.42
Dead	62.50	44.12	57.58

time_of_follow-up
Kruskal-Wallis test p-value: 0.0466021
Bonferroni adjusted p-value: 1

race
Chi-square test p-value: 0.02298358
Bonferroni adjusted p-value: 1

	mrHCC1	mrHCC2	mrHCC3
[Not Available]	8.33	0.98	0.00
[Not Evaluated]	0.00	1.96	0.00
[Unknown]	4.17	1.96	0.00
AMERICAN INDIAN OR ALASKA NATIVE	0.00	0.98	1.52
ASIAN	37.50	18.63	40.91
BLACK OR AFRICAN AMERICAN	4.17	7.84	7.58
WHITE	45.83	67.65	50.00

gender
Chi-square test p-value: 0.08767365
Bonferroni adjusted p-value: 1

	mrHCC1	mrHCC2	mrHCC3
FEMALE	33.33	27.45	43.94
MALE	66.67	72.55	56.06

age_at_initial_pathologic_diagnosis
Kruskal-Wallis test p-value: 0.001087563
Bonferroni adjusted p-value: 0.08809258

neoplasm_histologic_grade
Chi-square test p-value: 0.0036417
Bonferroni adjusted p-value: 0.2949777

	mrHCC1	mrHCC2	mrHCC3
[Not Available]	0.00	2.94	1.52
G1	8.33	26.47	7.58
G2	58.33	49.02	43.94
G3	33.33	21.57	46.97

residual_tumor
Chi-square test p-value: 0.2111647
Bonferroni adjusted p-value: 1

	mrHCC1	mrHCC2	mrHCC3
[Not Available]	4.17	4.90	1.52
R0	79.17	84.31	80.30
R1	8.33	3.92	7.58
R2	4.17	0.00	0.00
RX	4.17	6.86	10.61

pathologic_T
Chi-square test p-value: 0.2902317
Bonferroni adjusted p-value: 1

	mrHCC1	mrHCC2	mrHCC3
[Not Available]	0.00	0.98	0.00
T1	29.17	49.02	28.79
T2	29.17	19.61	31.82
T3	16.67	13.73	24.24
T3a	8.33	8.82	7.58
T3b	8.33	0.98	3.03
T4	8.33	5.88	4.55
TX	0.00	0.98	0.00

pathologic_N
Chi-square test p-value: 0.1504892
Bonferroni adjusted p-value: 1

	mrHCC1	mrHCC2	mrHCC3
[Not Available]	0.00	0.00	1.52
N0	58.33	62.75	66.67
N1	0.00	0.00	4.55
NX	41.67	37.25	27.27

pathologic_M
Chi-square test p-value: 0.04168974
Bonferroni adjusted p-value: 1

	mrHCC1	mrHCC2	mrHCC3
M0	79.17	67.65	78.79
M1	8.33	0.98	1.52
MX	12.50	31.37	19.70

pathologic_stage
Chi-square test p-value: 0.2454572
Bonferroni adjusted p-value: 1

	mrHCC1	mrHCC2	mrHCC3
[Not Available]	0.00	7.84	6.06
Stage I	29.17	48.04	25.76
Stage II	29.17	17.65	27.27
Stage III	0.00	1.96	1.52
Stage IIIA	25.00	19.61	27.27
Stage IIIB	4.17	1.96	4.55
Stage IIIC	4.17	1.96	4.55
Stage IV	4.17	0.00	1.52
Stage IVA	0.00	0.00	1.52
Stage IVB	4.17	0.98	0.00

Age>65
Chi-square test p-value: 0.02702547
Bonferroni adjusted p-value: 1

	mrHCC1	mrHCC2	mrHCC3
No	58.33	51.96	72.73
Yes	41.67	48.04	27.27

BMI
Kruskal-Wallis test p-value: 0.05395187
Bonferroni adjusted p-value: 1

ObesityClass1
Chi-square test p-value: 0.09237375
Bonferroni adjusted p-value: 1

	mrHCC1	mrHCC2	mrHCC3
---	4.17	12.75	16.67
#N/A	0.00	0.98	4.55
Normal	62.50	36.27	46.97
Obese	8.33	23.53	16.67
Overweight	25.00	26.47	15.15

ObesityClass2
Chi-square test p-value: 0.1322106
Bonferroni adjusted p-value: 1

	mrHCC1	mrHCC2	mrHCC3
---	4.17	12.75	16.67
#N/A	0.00	0.98	4.55
Normal	87.50	62.75	62.12
Obese	8.33	23.53	16.67

Asian Race
Chi-square test p-value: 0.001486905

Bonferroni adjusted p-value: 0.1204393

	mrHCC1	mrHCC2	mrHCC3
0	12.50	4.90	0.00
Asian	37.50	18.63	40.91
Not Asian	50.00	76.47	59.09

Caucasian
Chi-square test p-value: 0.003826918
Bonferroni adjusted p-value: 0.3099803

	mrHCC1	mrHCC2	mrHCC3
0	12.50	4.90	0.00
Caucasian	45.83	67.65	50.00
Not Caucasian	41.67	27.45	50.00

person_neoplasm_cancer_status
Chi-square test p-value: 0.6419603
Bonferroni adjusted p-value: 1

	mrHCC1	mrHCC2	mrHCC3
[Not Available]	8.33	2.94	4.55
[Unknown]	0.00	2.94	6.06
TUMOR FREE	66.67	65.69	57.58
WITH TUMOR	25.00	28.43	31.82

relative_family_cancer_history_ind_3
Chi-square test p-value: 0.414096
Bonferroni adjusted p-value: 1

	mrHCC1	mrHCC2	mrHCC3
[Not Available]	4.17	3.92	6.06
[Unknown]	8.33	15.69	9.09
NO	54.17	40.20	56.06
YES	33.33	40.20	28.79

Family History
Chi-square test p-value: 0.3180356
Bonferroni adjusted p-value: 1

	mrHCC1	mrHCC2	mrHCC3
---	12.50	19.61	15.15
No	54.17	40.20	56.06
Yes	33.33	40.20	28.79

history_hepato_carcinoma_risk_factor
Chi-square test p-value: 0.5731797
Bonferroni adjusted p-value: 1

	mrHCC1	mrHCC2
[Not Available]	0.00	0.98
[Unknown]	0.00	0.00
Alcohol consumption	25.00	25.49
Alcohol consumption|Hepatitis B	0.00	0.98
Alcohol consumption|Hepatitis B|Hepatitis C	0.00	0.98
Alcohol consumption|Hepatitis B|Hepatitis C|Hemochromatosis	0.00	0.98
Alcohol consumption|Hepatitis C	0.00	4.90
Alcohol consumption|Non-Alcoholic Fatty Liver Disease	0.00	2.94
Alcohol consumption|Other	4.17	0.98
Alpha-1 Antitrypsin Deficiency|Other	0.00	0.98
Hemochromatosis	0.00	0.00
Hepatitis B	12.50	7.84
Hepatitis B|Hepatitis C	0.00	0.98
Hepatitis B|Other	0.00	0.00
Hepatitis C	8.33	15.69
Hepatitis C|Other	0.00	0.00
No History of Primary Risk Factors	41.67	24.51
Non-Alcoholic Fatty Liver Disease	4.17	3.92
Non-Alcoholic Fatty Liver Disease|Other	0.00	0.98
Other	4.17	6.86

	mrHCC3
[Not Available]	7.58
[Unknown]	3.03
Alcohol consumption	22.73
Alcohol consumption|Hepatitis B	0.00
Alcohol consumption|Hepatitis B|Hepatitis C	0.00
Alcohol consumption|Hepatitis B|Hepatitis C|Hemochromatosis	0.00
Alcohol consumption|Hepatitis C	3.03
Alcohol consumption|Non-Alcoholic Fatty Liver Disease	0.00
Alcohol consumption|Other	1.52
Alpha-1 Antitrypsin Deficiency|Other	0.00
Hemochromatosis	3.03
Hepatitis B	10.61
Hepatitis B|Hepatitis C	0.00
Hepatitis B|Other	1.52
Hepatitis C	9.09
Hepatitis C|Other	1.52
No History of Primary Risk Factors	31.82
Non-Alcoholic Fatty Liver Disease	3.03
Non-Alcoholic Fatty Liver Disease|Other	0.00
Other	1.52

Alcoholic liver disease
Chi-square test p-value: 0.02611458
Bonferroni adjusted p-value: 1

	mrHCC1	mrHCC2	mrHCC3
---	4.17	0.98	12.12
Alcohol	29.17	37.25	27.27
No	66.67	61.76	60.61

Hepatitis C
Chi-square test p-value: 0.008433487
Bonferroni adjusted p-value: 0.6831124

	mrHCC1	mrHCC2	mrHCC3
---	4.17	0.98	12.12
Hepatitis C	8.33	23.53	13.64
No	87.50	75.49	74.24

Hepatitis B
Chi-square test p-value: 0.03630778
Bonferroni adjusted p-value: 1

	mrHCC1	mrHCC2	mrHCC3
---	4.17	0.98	12.12
Hepatitis B	12.50	11.76	12.12
No	83.33	87.25	75.76

NAFLD
Chi-square test p-value: 0.0174004
Bonferroni adjusted p-value: 1

	mrHCC1	mrHCC2	mrHCC3
---	0.00	0.98	10.61
NAFLD	4.17	7.84	3.03
No	95.83	91.18	86.36

Type of macrovascular invasion
Chi-square test p-value: 0.03532173
Bonferroni adjusted p-value: 1

	mrHCC1	mrHCC2	mrHCC3
---	16.67	14.71	22.73
Macrovascular Invasion	8.33	0.00	9.09
Microvascular	29.17	22.55	22.73
None	45.83	62.75	45.45

Vascular Invasion
Chi-square test p-value: 0.1794046
Bonferroni adjusted p-value: 1

	mrHCC1	mrHCC2	mrHCC3
---	16.67	14.71	22.73
No	45.83	62.75	45.45
Yes	37.50	22.55	31.82

child_pugh_classification_grade
Chi-square test p-value: 0.0340016

Bonferroni adjusted p-value: 1

	mrHCC1	mrHCC2	mrHCC3
[Not Available]	8.33	17.65	31.82
[Unknown]	20.83	21.57	28.79
A	62.50	51.96	28.79
B	8.33	8.82	10.61

alpha_fetoprotien_at_procurement
Kruskal-Wallis test p-value: 0.3052903
Bonferroni adjusted p-value: 1

AFP>300
Chi-square test p-value: 0.0003467845
Bonferroni adjusted p-value: 0.02808955

	mrHCC1	mrHCC2	mrHCC3
	0.00	0.00	0.00
No	63.16	90.54	60.98
Yes	36.84	9.46	39.02

liver_fibrosis_ishak_score_category
Chi-square test p-value: 0.02579391
Bonferroni adjusted p-value: 1

	mrHCC1	mrHCC2	mrHCC3
[Not Available]	16.67	13.73	36.36
[Unknown]	4.17	0.98	0.00
0 - No Fibrosis	54.17	37.25	28.79
1,2 - Portal Fibrosis	8.33	13.73	7.58
3,4 - Fibrous Speta	8.33	5.88	9.09
5 - Nodular Formation and Incomplete Cirrhosis	0.00	1.96	3.03
6 - Established Cirrhosis	8.33	26.47	15.15

Cirrhosis
Chi-square test p-value: 0.1126564
Bonferroni adjusted p-value: 1

	mrHCC1	mrHCC2	mrHCC3
---	8.33	2.94	1.52
No	83.33	67.65	77.27
Yes	8.33	29.41	21.21

new_tumor_event_after_initial_treatment
Chi-square test p-value: 0.1053778
Bonferroni adjusted p-value: 1

	mrHCC1	mrHCC2	mrHCC3
[Not Available]	62.50	32.35	37.88
[Unknown]	0.00	0.98	6.06
NO	20.83	31.37	28.79
Yes	0.00	0.98	0.00

YES	16.67	34.31	27.27

Hepatitis Calls

HBV_consensus
Chi-square test p-value: 0.1571745
Bonferroni adjusted p-value: 1

	mrHCC1	mrHCC2	mrHCC3
neg	75.00	82.35	69.70
pos	25.00	17.65	30.30

HCV_consensus
Chi-square test p-value: 0.1089897
Bonferroni adjusted p-value: 1

	mrHCC1	mrHCC2	mrHCC3
neg	91.67	76.47	86.36
pos	8.33	23.53	13.64

Leukocyte Estimate

Leukocyte_estimate
Kruskal-Wallis test p-value: 0.004087824
Bonferroni adjusted p-value: 0.3311137

Purity/Ploidy

Purity
Kruskal-Wallis test p-value: 0.0003424982
Bonferroni adjusted p-value: 0.02774235

Ploidy
Kruskal-Wallis test p-value: 0.0005763198
Bonferroni adjusted p-value: 0.04668191

Genome_Doublings
Chi-square test p-value: 1.528364e-05
Bonferroni adjusted p-value: 0.001237975

	mrHCC1	mrHCC2	mrHCC3
0	42.86	76.60	43.55
1	19.05	18.09	33.87
2	38.10	5.32	22.58

Cancer_DNA_fraction
Kruskal-Wallis test p-value: 0.02205458
Bonferroni adjusted p-value: 1

Historic Gene Signature

NCIP
Chi-square test p-value: 8.17942e-19
Bonferroni adjusted p-value: 6.62533e-17

	mrHCC1	mrHCC2	mrHCC3
A	54.17	5.88	72.73
B	45.83	94.12	27.27

NCIHS
Chi-square test p-value: 5.895402e-07
Bonferroni adjusted p-value: 4.775276e-05

	mrHCC1	mrHCC2	mrHCC3
HC	91.67	96.08	66.67
HS	8.33	3.92	33.33

NCIPHS
Chi-square test p-value: 1.893813e-18
Bonferroni adjusted p-value: 1.533989e-16

	mrHCC1	mrHCC2	mrHCC3
A	47.83	4.95	37.88
B	43.48	83.17	13.64
HS	8.70	11.88	48.48

SNUR
Chi-square test p-value: 2.846825e-12
Bonferroni adjusted p-value: 2.305928e-10

	mrHCC1	mrHCC2	mrHCC3
High	33.33	15.69	71.21
Low	66.67	84.31	28.79

HB16
Chi-square test p-value: 4.474791e-19
Bonferroni adjusted p-value: 3.624581e-17

	mrHCC1	mrHCC2	mrHCC3
C1	62.50	92.16	22.73
C2	37.50	7.84	77.27

RS65
Chi-square test p-value: 2.368049e-15
Bonferroni adjusted p-value: 1.91812e-13

	mrHCC1	mrHCC2	mrHCC3
high	41.67	6.86	66.67

low	58.33	93.14	33.33

RS65.Score
Kruskal-Wallis test p-value: 1.187344e-20
Bonferroni adjusted p-value: 9.617487e-19

Hoshida
Chi-square test p-value: 1.384376e-21
Bonferroni adjusted p-value: 1.121345e-19

	mrHCC1	mrHCC2	mrHCC3
1	20.83	16.67	9.09
2	70.83	11.76	84.85
3	8.33	71.57	6.06

HIPPO
Chi-square test p-value: 1.585921e-06
Bonferroni adjusted p-value: 0.0001284596

	mrHCC1	mrHCC2	mrHCC3
AH	66.67	91.18	57.58
SOH	33.33	8.82	42.42

CC-like
Chi-square test p-value: 4.450136e-18
Bonferroni adjusted p-value: 3.60461e-16

	mrHCC1	mrHCC2	mrHCC3
CCL	33.33	2.94	66.67
HCC	66.67	97.06	33.33

IDH
Chi-square test p-value: 0.0981672
Bonferroni adjusted p-value: 1

	mrHCC1	mrHCC2	mrHCC3
IDH mutant	4.17	0.00	4.55
WT	95.83	100.00	95.45

IDH_P
Kruskal-Wallis test p-value: 1.309825e-08
Bonferroni adjusted p-value: 1.060958e-06

TP53 / CTNNB1 / TERT

TP53_mutation
Chi-square test p-value: 0.01301742
Bonferroni adjusted p-value: 1

	mrHCC1	mrHCC2	mrHCC3
mut	26.09	23.23	44.62
wt	73.91	76.77	55.38

TP53 mutation signature (sum of expression of targets)
Kruskal-Wallis test p-value: 1.032032e-10
Bonferroni adjusted p-value: 8.359459e-09

CTNNB1_mutation
Chi-square test p-value: 0.006422675
Bonferroni adjusted p-value: 0.5202367

	mrHCC1	mrHCC2	mrHCC3
mut	30.43	34.34	12.31
wt	69.57	65.66	87.69

TERT promoter mutation
Chi-square test p-value: 0.2431761
Bonferroni adjusted p-value: 1

	mrHCC1	mrHCC2	mrHCC3
mut	41.67	50.98	34.85
no	54.17	48.04	63.64
not assessed	4.17	0.98	1.52

TERT mRNA expression
Kruskal-Wallis test p-value: 0.4653658
Bonferroni adjusted p-value: 1

Pathology Review

HCC subtypes
Chi-square test p-value: 0.005754126
Bonferroni adjusted p-value: 0.4660842

	mrHCC1	mrHCC2	mrHCC3
Cirrhotomimetic hepatocellular carcinoma	5.00	0.00	3.12
Clear cell hepatocellular carcinoma	0.00	8.42	4.69
Fibrolamellar carcinoma	0.00	0.00	6.25
Lymphocyte rich hepatocellular carcinoma	0.00	0.00	4.69
Myxoid hepatocellular carcinoma	0.00	1.05	0.00
No specific subtype	80.00	82.11	67.19
Sarcomatoid hepatocellular carcinoma	0.00	0.00	3.12
Scirrhous hepatocellular carcinoma	15.00	1.05	6.25
Steatohepatitic	0.00	7.37	4.69

Grade-predominant
Chi-square test p-value: 0.0008726526
Bonferroni adjusted p-value: 0.07068486

	mrHCC1	mrHCC2	mrHCC3
1	0.00	4.21	0.00
2	10.00	35.79	10.94
3	75.00	49.47	64.06
4	15.00	10.53	25.00

Grade-worst
Chi-square test p-value: 0.00263232
Bonferroni adjusted p-value: 0.2132179

	mrHCC1	mrHCC2	mrHCC3
1	0.00	2.11	0.00
2	5.00	23.16	4.69
3	65.00	53.68	50.00
4	30.00	21.05	45.31

Clear Cell Change
Kruskal-Wallis test p-value: 0.8620273
Bonferroni adjusted p-value: 1

Intratumoral hyaline bodies
Kruskal-Wallis test p-value: 0.4482744
Bonferroni adjusted p-value: 1

Intratumoral Mallory hyaline bodies
Kruskal-Wallis test p-value: 0.1408251
Bonferroni adjusted p-value: 1

Macrovesicular steatosis
Kruskal-Wallis test p-value: 0.3084989
Bonferroni adjusted p-value: 1

Ballooned tumor cells
Chi-square test p-value: 0.2824133
Bonferroni adjusted p-value: 1

	mrHCC1	mrHCC2	mrHCC3
No	85.00	75.79	82.81
Yes Few	10.00	11.58	14.06
Yes Many	5.00	12.63	3.12

Intratumoral inflammation
Chi-square test p-value: 0.29864
Bonferroni adjusted p-value: 1

	mrHCC1
None or minimal	80.00
Yes, lymphocytic Marked (on average, more inflammatory cells than tumor)	0.00
Yes, lymphocytic Mild	15.00
Yes, lymphocytic Moderate	5.00
Yes, neutrophilic Mild	0.00

	mrHCC2
None or minimal	84.21
Yes, lymphocytic Marked (on average, more inflammatory cells than tumor)	0.00
Yes, lymphocytic Mild	14.74

Yes, lymphocytic Moderate	1.05
Yes, neutrophilic Mild	0.00

	mrHCC3
None or minimal	71.88
Yes, lymphocytic Marked (on average, more inflammatory cells than tumor)	3.12
Yes, lymphocytic Mild	17.19
Yes, lymphocytic Moderate	6.25
Yes, neutrophilic Mild	1.56

Cholestasis
Chi-square test p-value: 0.5459086
Bonferroni adjusted p-value: 1

	mrHCC1	mrHCC2	mrHCC3
No	65.00	70.53	79.69
Yes Mild (less than 5% of tumor area)	25.00	24.21	17.19
Yes Moderate (6-50% of tumor area)	10.00	5.26	3.12

Intratumoral fibrosis
Chi-square test p-value: 0.04370329
Bonferroni adjusted p-value: 1

	mrHCC1
None or minimal	70.00
Yes Marked (fibrosis is equal to or greater than the amount of tumor cells)	5.00
Yes Mild (intratumoral fibrosis less than 5-25% of surface area)	10.00
Yes Moderate (intratumoral fibrosis 26- 49% of surface area)	15.00

	mrHCC2
None or minimal	84.21
Yes Marked (fibrosis is equal to or greater than the amount of tumor cells)	0.00
Yes Mild (intratumoral fibrosis less than 5-25% of surface area)	12.63
Yes Moderate (intratumoral fibrosis 26- 49% of surface area)	3.16

	mrHCC3
None or minimal	67.19
Yes Marked (fibrosis is equal to or greater than the amount of tumor cells)	1.56
Yes Mild (intratumoral fibrosis less than 5-25% of surface area)	23.44
Yes Moderate (intratumoral fibrosis 26- 49% of surface area)	7.81

Growth pattern-major
Chi-square test p-value: 0.0001915865
Bonferroni adjusted p-value: 0.0155185

	mrHCC1	mrHCC2	mrHCC3
Macrotrabecular	40.00	3.16	10.94
Pseudoacinar	10.00	9.47	6.25
Solid	20.00	44.21	45.31
Trabecular	30.00	43.16	37.50

Growth pattern-other
Chi-square test p-value: 0.3760001
Bonferroni adjusted p-value: 1

	mrHCC1	mrHCC2	mrHCC3
Macrotrabecular	0.00	1.05	1.56
Macrotrabecular Solid	0.00	0.00	3.12
Macrotrabecular Trabecular	0.00	0.00	1.56
NULL	5.00	13.68	14.06
Pseudoacinar	5.00	7.37	1.56
Pseudoacinar Macrotrabecular	0.00	2.11	1.56
Pseudoacinar Macrotrabecular Solid	0.00	0.00	1.56
Pseudoacinar Solid	5.00	1.05	4.69
Pseudoacinar Solid Macrotrabecular	0.00	1.05	0.00
Pseudoacinar Trabecular	10.00	8.42	6.25
Pseudoacinar Trabecular Macrotrabecular	0.00	0.00	3.12
Solid	15.00	16.84	12.50
Solid Macrotrabecular	0.00	3.16	4.69
Solid Macrotrabecular Pseudoacinar	0.00	3.16	0.00
Solid Pseudoacinar	5.00	7.37	6.25
Solid Pseudoacinar Trabecular	0.00	0.00	0.00
Solid Trabecular	10.00	3.16	4.69
Solid Trabecular Pseudoacinar	5.00	0.00	1.56
Trabecular	10.00	21.05	17.19
Trabecular Macrotrabecular	5.00	1.05	7.81
Trabecular Macrotrabecular Pseudoacinar	0.00	1.05	0.00
Trabecular Pseudoacinar	10.00	5.26	0.00
Trabecular Solid	15.00	3.16	6.25

Multimorphologies
Chi-square test p-value: 0.682271
Bonferroni adjusted p-value: 1

	mrHCC1
No	95.00
Yes - Clearly distinct nodules of HCC with different morphological patterns	5.00

	mrHCC2
No	88.42
Yes - Clearly distinct nodules of HCC with different morphological patterns	11.58

	mrHCC3
No	89.06
Yes - Clearly distinct nodules of HCC with different morphological patterns	10.94

mRNA Fusions

mRNA fusions
Chi-square test p-value: 0.5036021
Bonferroni adjusted p-value: 1

	mrHCC1	mrHCC2	mrHCC3
---	95.83	89.22	83.33
ARID1A-PRKCZ	0.00	0.98	0.00
CTSC-RAB38	0.00	0.00	3.03
DNAJB1/PRKACA	0.00	0.00	6.06
FGFR2-BICC1	0.00	0.00	1.52
LOC283587-TERT	0.00	0.98	0.00
PSPC-FGF14	4.17	0.00	0.00
PTK2-DENND3	0.00	0.98	0.00
SLC12A7-TERT	0.00	0.98	1.52
SLC7A2-TERT	0.00	0.98	0.00
STK38-TDRD7	0.00	0.98	0.00
STRN3-MAML3	0.00	0.00	1.52
TACC3-FGFR3; TCF7L2-RP11-202P11.1	0.00	0.98	0.00
TCF7L2-VTI1A	0.00	0.98	0.00
TCF7L2-VTI1A; EP300-RBX1	0.00	0.98	0.00
TFG-GPR128	0.00	0.00	1.52
XRCC5-ABCB4; RAD50-C5orf56	0.00	0.98	0.00
XRCC6-ACO2	0.00	0.98	1.52
